# Supplementary material for: A Piezoelectric MEMS Speaker with a Combined Function of a Silent Alarm
Source: Micromachines (Basel). 2023 Mar 22;14(3):702. doi: 10.3390/mi14030702 (PMC10057705; doi:10.3390/mi14030702)
Supplement: Supplementary file 1 [file micromachines-14-00702-s001.zip › micromachines-2255962-supplementary.pdf]

Piezoelectric MEMS Speaker Combining Function of Silent Alarm

Qi Wang[1,2], Tao Ruan[1,2], Qingda Xu[1,2], Zhiyong Hu[1,2], Bin Yang[1], Minmin You[1], Zude Lin[1], and Jingquan Liu[1*]*

[1]National Key Laboratory of Science and Technology on Micro/Nano Fabrication, Shanghai Jiao Tong University, Shanghai, 200240, China

[2]Collaborative Innovation Center of IFSA, Department of Micro/Nano-electronics, Shanghai Jiao Tong University, Shanghai, 200240, China

[*] Corresponding author.

E-mail address: linzude@sjtu.edu.cn; jqliu@sjtu.edu.cn

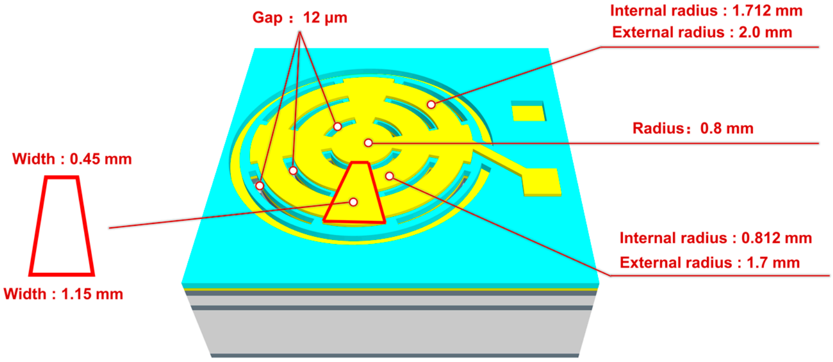

Figure S1. Detailed sizes of device.

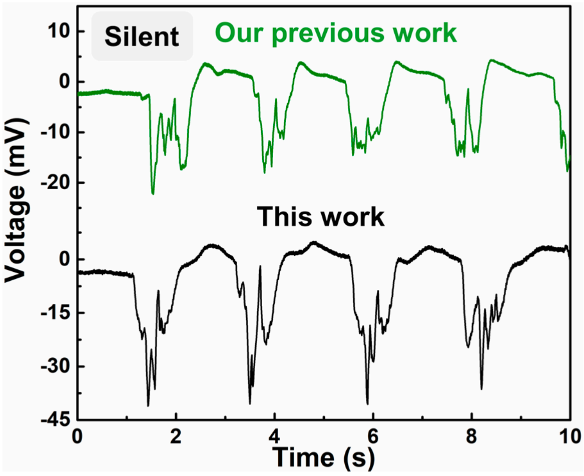

Figure S2. Comparison of voltage signals between this work and our previous work.

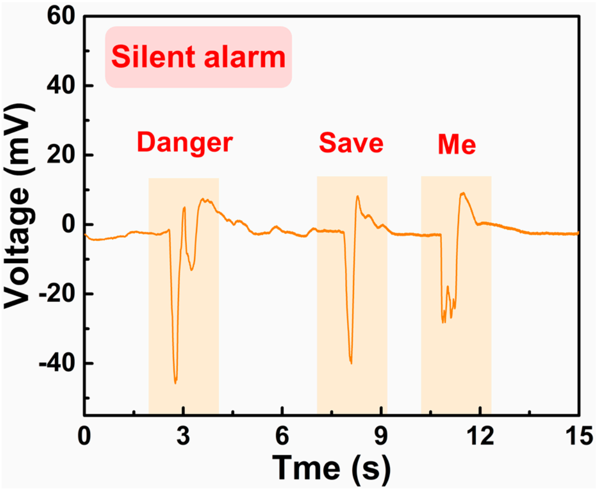

Figure S3. Silent alarm in dangerous situations.
